# How can Big Data Analytics Support People-Centred and Integrated Health Services: A Scoping Review

**DOI:** 10.5334/ijic.5543

**Published:** 2022-06-16

**Authors:** Timo Schulte, Sabine Bohnet-Joschko

**Affiliations:** 1 Witten/Herdecke University, Germany

**Keywords:** Big Data, people-centred and integrated health services, advanced analytics, Personal Health Record, health platform, machine Learning

## Abstract

**Introduction:**

Health systems in high-income countries face a variety of challenges calling for a systemic approach to improve quality and efficiency. Putting people in the centre is the main idea of the WHO model of people-centred and integrated health services. Integrating health services is fuelled by an integration of health data with great potentials for decision support based on big data analytics. The research question of this paper is “How can big data analytics support people-centred and integrated health services?”

**Methods:**

A scoping review following the recommendations of the Preferred Reporting Items for Systematic Reviews and Meta-Analyses – Scoping Review (PRISMA-ScR) statement was conducted to gather information on how big data analytics can support people-centred and integrated health services. The results were summarized in a role model of a people-centred and integrated health services platform illustrating which data sources might be integrated and which types of analytics might be applied to support the strategies of the people-centred and integrated health services framework to become more integrated across the continuum of care. Additional rapid literature reviews were conducted to generate frequency distributions of the most often used data types and analytical methods in the medical literature. Finally, the main challenges connected with big data analytics were worked out based on a content analysis of the results from the scoping literature review.

**Results:**

Based on the results from the rapid literature reviews the most often used data sources for big data analytics (BDA) in healthcare were biomarkers (39.3%) and medical images (30.9%). The most often used analytical models were support vector machines (27.3%) and neural networks (20.4%). The people-centred and integrated health services framework defines different strategic interventions for health services to become more integrated. To support all aspects of these interventions a comparably integrated platform of health-related data would be needed, so that a role model labelled as people-centred health platform was developed. Based on integrated data the results of the scoping review (n = 72) indicate, that big data analytics could for example support the strategic intervention of tailoring personalized health plans (43.1%), e.g. by predicting individual risk factors for different therapy options. Also BDA might enhance clinical decision support tools (31.9%), e.g. by calculating risk factors for disease uptake or progression. BDA might also assist in designing population-based services (26.4% by clustering comparable individuals in manageable risk groups e.g. mentored by specifically trained, non-medical professionals. The main challenges of big data analytics in healthcare were categorized in regulatory, (information-) technological, methodological, and cultural issues, whereas methodological challenges were mentioned most often (55.0%), followed by regulatory challenges (43.7%).

**Discussion:**

The BDA applications presented in this literature review are based on findings which have already been published. For some important components of the framework on people-centred care like enhancing the role of community care or establishing intersectoral partnerships between health and social care institutions only few examples of enabling big data analytical tools were found in the literature. Quite the opposite does this mean that these strategies have less potential value, but rather that the source systems in these fields need to be further developed to be suitable for big data analytics.

**Conclusions:**

Big data analytics can support people-centred and integrated health services e.g. by patient similarity stratifications or predictions of individual risk factors. But BDA fails to unfold its full potential until data source systems are still disconnected and actions towards a comprehensive and people-centred health-related data platform are politically insufficiently incentivized. This work highlighted the potential of big data analysis in the context of the model of people-centred and integrated health services, whereby the role model of the person-centered health platform can be used as a blueprint to support strategies to improve person-centered health care. Likely because health data is extremely sensitive and complex, there are only few practical examples of platforms to some extent already capable of merging and processing people-centred big data, but the integration of health data can be expected to further proceed so that analytical opportunities might also become reality in the near future.

## Abstract (de)

**Einleitung:**

Gesundheitssysteme in entwickelten Ländern stehen vor vielen Herausforderungen in Bezug auf die Verbesserung von Qualität und Effizienz, die zum Teil nur durch Veränderungen auf der Systemebene bewältigt werden können. Die Versorgung im Einklang mit den Bedürfnissen von Personen zu gestalten ist der zentrale Gedanke des WHO-Modells der personen-zentrierten und integrierten Versorgung (*people-centred and integrated health services*). Die Integration der Gesundheitsversorgung wird zusätzlich durch die Integration von Gesundheitsdaten mit enormen Potenzialen zur Entscheidungsunterstützung durch die Analyse von Big Data vorangetrieben. Die Forschungsfrage dieser Arbeit ist „Wie kann die Analyse von Big Data eine personenzentrierte und integrierte Gesundheitsversorgung unterstützen?“

**Methoden:**

Es wurde ein Scoping Review gemäß „Preferred Reporting Items for Systematic Reviews and Meta-Analyses – Scoping Review (PRISMA-ScR)“ durchgeführt, um Informationen zusammenzutragen, wie Big Data Analysen eine personen-zentrierte und integrierte Versorgung unterstützen können. Sämtliche Ergebnisse wurden in einem Modell einer personen-zentrierten und integrierten Gesundheitsplattform zusammengefasst, die veranschaulicht, welche Datenquellen integriert und welche analytischen Modelle darauf angewendet werden könnten, um Strategien zu unterstützen, die darauf ausgerichtet sind, eine personen-zentrierte Versorgung voranzutreiben. Übersichtsartige Rapid Reviews wurden genutzt, um Häufigkeitsverteilungen der zum Zeitpunkt der Durchführung dieses Reviews am häufigsten verwendeten Datentypen und analytischen Methoden zur Analyse von Big Data in der medizinischen Literatur zu erstellen. Außerdem wurden die am häufigsten erwähnten Herausforderungen im Zusammenhang mit der Analyse von Big Data im Gesundheitswesen auf Basis einer Inhaltsanalyse der Artikel aus dem Scoping Review herausgearbeitet.

**Ergebnisse:**

Basierend auf den Ergebnissen der Rapid Reviews sind die derzeit am häufigsten genutzten Datenquellen für Big Data Analysen (BDA) im Gesundheitswesen genetische Biomarker-Daten (39,3%) und Daten der medizinischen Bildgebung (30,9%). Die am häufigsten genutzten analytischen Methoden sind Support Vector Machines (27,3%) und neurale Netzwerke (20,4%). Das WHO-Modell der personenzentrierten und integrierten Versorgung definiert verschiedene strategische Interventionen. Um sämtliche Aspekte dieser Interventionen unterstützen zu können, sollte die Datenintegration ähnlich umfassend ausfallen. Das im Zuge dieser Publikation entwickelte Modell der Integration von Gesundheitsdaten wird als „personen-zentrierte Gesundheitsplattform“ bezeichnet. Die Ergebnisse des Scoping Reviews legen nahe, dass, sofern ein Zugriff auf integrierte Daten möglich ist, die darauf basierenden BDA genutzt werden könnten, um (bessere) individualisierte Gesundheitspläne zu erstellen (43,1%), z.B. durch Prädiktion persönlicher Risikofaktoren in Bezug auf verschiedene Therapieoptionen. Außerdem könnten BDA klinische Systeme der Entscheidungsunterstützung erweitern und verbessern (31,9%), z.B. durch die Berechnung von Risikofaktoren für Krankheitsentstehung oder -entwicklung. Zusätzlich könnten BDA hilfreich für die Ausgestaltung populations-basierter Modelle der Gesundheitsversorgung (26,4%) sein, etwa durch Kategorisierung von Personen in vergleich- und steuerbare Risikogruppen, die u.a. von speziell ausgebildeten, nicht-medizinischen Spezialisten betreut werden könnten. Die am häufigsten erwähnten Herausforderungen im Zusammenhang mit BDA im Gesundheitswesen wurden in regulatorisch-gesetzgeberische, (informations-)technologische, methodische und kulturelle Aspekte kategorisiert, wobei die methodischen Herausforderungen am häufigsten erwähnt wurden (55,0%), gefolgt von regulatorischen Herausforderungen (43,7%).

**Diskussion:**

Die Ergebnisse dieses Reviews basieren ausschließlich auf Erkenntnissen, die bereits veröffentlicht wurden. Für einige wichtige Komponenten des WHO-Modell einer personen-zentrierten Versorgung wie z.B. der verstärkten Bedeutung wohnortnaher Versorgungsangebote oder der Etablierung von Kooperationen zwischen Institutionen der Gesundheits- und Sozialversorgung wurden nur wenige Beispiele in der Literatur gefunden, wie diese Aspekte durch die Auswertung von Big Data verbessert werden könnten. Dies soll nicht den Anschein erwecken, dass diese Interventionen einen geringeren strategischen Wert haben, sondern es ist eher ein Indiz dafür, dass die Datenquellen in diesen Bereichen noch weiterentwickelt und integriert werden müssen, um Big Data Analytics zu ermöglichen.

**Schlussfolgerung:**

BDA können die Interventionen einer personen-zentrierten und integrierten Versorgung unterstützen, z.B. durch Risikostratifizierung oder durch Prädiktion individueller Erfolgs- und Risikofaktoren. Aber derzeit sind BDA noch nicht in der Lage ihr volles Potenzial zu entfalten, da die Quellsysteme von Gesundheitsdaten größtenteils nicht verknüpft bzw. integriert sind und politisch zu wenig Anreize für die Entwicklung personen-zentrierter Gesundheitsplattformen gesetzt werden. Diese Arbeit beleuchtete die Potenziale von Big Data Analysen im Zusammenhang mit dem Modell einer personenzentrierten Gesundheitsversorgung, wobei das Modell der personen-zentrierten Gesundheitsplattform als Blaupause zur Unterstützung von Strategien zur Verbesserung personenzentrierter Gesundheitsversorgung genutzt werden kann. Aufgrund der Komplexität und Sensibilität von Gesundheitsdaten gibt es nur wenige Beispiele von Gesundheitsplattformen, die bereits sämtliche Typen von personenzentrierten Gesundheitsdaten für Big Data Analysen integrieren, aber es darf erwartet werden, dass die Integration von Gesundheitsdaten weiter voranschreiten wird, so dass auch die analytischen Anwendungen in naher Zukunft eine echte Unterstützung der Versorgungsrealität werden können.

## Introduction

Despite differing institutional arrangements, health systems in developed countries face a variety of similar challenges including financial constraints, a rising demand for health services due to demographic changes, increasing multi-morbidity and unhealthy behaviours as well as growing expectations of citizens [1]. These challenges arise from and are reinforced by misaligned financing and highly fragmented processes of health care delivery [2]. To meet these challenges, there is a need for a systemic approach to improve treatment processes focusing on improvements of quality and efficiency [3,4]. Transformation toward value-based healthcare is accompanied by a change in focus from provider-centred models, with a lack of coordination across sectors, to more patient-centred models of healthcare delivery [5] as described in the people-centred and integrated health services (PCIHS) framework [6]. Putting people rather than providers or diseases in the centre, PCIHS will foster people-centred models of data integration and vice versa will progresses in computational storage and processing power [7] as well as accelerating adoptions of electronic data sources facilitate health service integration [8,9,10,11] and support activities towards the triple aim [12,13]. The emerging data sets and advanced analytical capabilities are believed to be part of the most important innovations in healthcare [14,15].

The research question “How can big data analytics support people-centred and integrated health services?” was investigated by performing a scoping literature review. Big data analytical applications which might act as enablers to the five strategical domains proposed by the WHO for health services to become more integrated and people-centred were thereby worked out. To the best of the authors’ knowledge a combination of the concepts of PCIHS and big data analytics (BDA) was not presented in any previous publication. The estimation, that transforming the already existing big data assets into actionable knowledge could reduce costs only in the healthcare system of the USA by $300 to $450 billion per year [16] demonstrates the potential impact of BDA. The results presented in this work might be helpful for health policy in reinventing health systems as well as for providers and other healthcare decision makers struggling to work collaboratively within the context of their health systems.

## Materials and methods

At first some key terms will be briefly defined before describing the methodology of the scoping literature review and the additional rapid literature reviews.

### People-centred, integrated health services (PCIHS)

Designing health services in accordance with the determinants of health spanning biophysical, lifestyle-related, social, health system-related, and environmental factors challenges traditional disease-centred, fragmented models of health service delivery [17,18]. In response to the challenges in healthcare, different concepts of integrated care emerged, centred on the needs of patients, their families, and their communities [19]. The concepts vary in size and scope and are designed around the idea to put people in the centre of service delivery to improve value-creation [3,20]. Several of these approaches including the rainbow model of care were considered when researches designed the framework for people-centred and integrated health services (PCIHS) for the World Health Organization (WHO) [18]. In the WHO’s global vision, not only does it outline achieving a seamless patient experience but also focusing on health promotion and disease prevention for the people, which may not necessarily be patients yet [2]. Improving healthcare following this people-centred perspective must focus on all the potential interrelations of the determinants of health and uniting the diverse objectives of healthcare stakeholders [21,22,23,24,25,26] across the continuum of health promotion, disease prevention, disease detection and acute, chronic, and palliative care [24,25,27,28,29].

The PCIHS framework proposes five strategies for health services to become more integrated [30,31,32]:

- Empowering and engaging people and communities(e.g. personalized health plans, shared decision making, access to health records)
- Strengthening governance and accountability(e.g. acting upon user experience, decentralization, performance evaluation)
- Reorienting the model of care(e.g. strengthening primary and community care, population health, prevention)
- Coordinating services within and across sectors(e.g. care coordination, effective referral and discharge systems, coordinated systems)
- Creating an enabling environment(e.g. large scale systems change, strong leadership, financial support, cultural change)

### Big data

Although a consensus about the definition does not exist, it can be agreed upon that massive data storage alone does not define big data [27,33]. The definition referenced most often is rooting in the 3-V model focusing on the characteristics of volume, velocity, and variety [34], which was gradually enhanced to the 5-V model by adding veracity and value [14,35,36,37,38,39,40]. Accordingly big data is characterized by

**Table:** 

•	high volume	(big amount of data, often referred to as exceeding tera- or petabytes),
•	high velocity	(fast speed of data generation like streaming data close to real-time),
•	high variety	(many diverse data formats and structures from multiple sources),
•	high veracity	(conformity with facts and closely related to data quality),
•	high value	(the information derived provides benefits to decision makers which in healthcare is closely related to the triple aim).

### Big data types in healthcare

The fragmentation of patient care is also reflected in the decentralization of health data [41,42]. In general, any source contributing information to one of the factors influencing people’s health can be valuable [22], although not all data types abide by all criteria of the 5V-model. The most common types data in healthcare are billing data, clinical data, patient- or people-generated data, health-related research data and data collected externally to the health care environment including socio-economical, societal, community-based, demographical, environmental, and other health-related data (see Table 1) [27,43,44].

**Table 1:** Data types for big data analytics in healthcare by data generation point.


DATA GENERATION POINTS	DATA TYPES	EXAMPLES ON TYPICAL DATA CONTENT

Transactions/billing with different payer organizations	Administrative data	Patient demographics, plan types, type of provider, location, …

	Medical claims	In-/outpatient visits, diagnosis/procedure coding, referrals, …

	Pharmaceutical claims	Drug codes, dosages, prescription dates, manufacturer, …

	Ancillary claims	Medical equipment, physiotherapy, home health assistance, …

Clinical/diagnostic processes of different provider organizations (e.g., health, social, aged or disability care)	Institutional data	Educational background, work experience, working times, …

	EMR/EHR data	Vital signs, medical history, disease conditions, lab results, …

	Medical imaging	X-ray, magnetic resonance, computed tomography, ultrasonography, …

	Biomarker	“-omics”: genomics, proteomics, metabolomics, lipidomics, …

	Registries	Structured collection of disease/population specific measures

Patient- or people-generated	Smart sensor/device data	Biometric data, physical activity, gait/sleep patterns, location, …

	Web usage data	Social media posts, internet search logs, health forum activity, …

Health-related research	Clinical trial data	Study size, clinically defined parameters and outcomes, …

	Drug surveillance data	Adverse drug effects, population size, regional uptake/variation, …

	(Health) Survey data	Patient-reported outcome measures (PROMs), health literacy, …

Health-related systems	Socio-economic/community-based data	Income, deprivation, education, living situation, marital status, …

	Environmental/spatial data	Air/noise pollution, temperature, neighbourhood characteristics, …


A good overview on sources, stakeholders and capabilities in the health data ecosystem is provided by Vayena et al. [45].

### Big data analytics (BDA) in healthcare

For big data analytics there is also no consented definition. Compliant to other industries analytical types in healthcare [38,46,47] can be categorized in

- descriptive analytics (What happened or is happening?),
- predictive analytics (What is likely to happen next?),
- explorative analytics (Why is it happening? What is unknown yet?),
- prescriptive analytics (Which decision is best to reach a desired outcome?).

From a methodological perspective the terms “prediction” and “exploration” do not define different approaches, but different analytical purposes [48]. Taken together predictive and explorative analytics are also referred to as advanced analytics [49]. Performing advanced analytics on big data is one approach to define big data analytics (BDA) [14,15]. In a broader sense all kinds of predictive or explorative models applied to big data would meet this definition, also including statistical methods [50] and most often when the aspect of high velocity is inconclusively. In a narrower sense only inductive approaches like data mining or machine learning suited for high-dimensional data sets define big data analytics [10,27,46,51,52]. For this paper the broader focus was adapted. Big data analytics (BDA) can provide complementary information to those derived from hypothesis-based experiments which have a long tradition in healthcare [46,51,53,54,55]. As there is plenty of literature on statistical methods they are not further explained (see e.g., Hohmann et al. [56]). Machine learning has the potential to enhance statistical analytics by providing models that allow for more multivariate effects and complex relationships. While supervised learning is used to train algorithms in predictions, unsupervised learning is used for exploring unknown patterns within data sets [7,57], whereas the analytical methods are basically the same as for both tasks [48,58].

#### Machine learning models

Supervised machine learning encompasses hypothesis-free algorithms which do not need assumptions about the data distribution. Furthermore, an inclusion of high-dimensional and highly correlated input variables is often appropriate for model optimization [36,56]. In course of supervised learning the target variable has to be (human-)labelled and the prediction is deducted normally based on three stages in a causal chain: training, validation and testing [56,59]. To train the model it analyses a set of observations to identify discriminating features of the predictor variable and performs optimization algorithms to reproduce the outcome [38,60].

#### Unsupervised machine learning models

Unsupervised learning algorithms are not provided with human labelled target variables and leave the probability of the input variables undefined [48]. They search for the most frequent simultaneous occurrence of certain (patient) characteristics not having a potential structure or hypothesis in mind [61]. By using unspecified criteria cohorts are not necessarily disease-derived but feature-derived enabling dynamic risk groups [22]. The algorithms shall separate low dimensional, unlabelled samples to find a hidden structure represented by the deduction of as many reasonable distinctive classes as possible [7]. Humans are normally reintegrated during the process of data interpretation, which is supported by visualizing the results using graphical models [62,63].

### Review method and content analysis

To first of all provide a comparative overview on the “data types” and “analytical methods” used most often in healthcare, rapid literature reviews were conducted in *Medline/PubMed* combining the search terms of the scoping review described in the following with terms specifying the data types and analytical models (see Table 4 and Table 5 in the appendix).

To answer the main research question “How can big data analytics support people-centred and integrated health services?” a scoping review following the recommendations of the *Preferred Reporting Items for Systematic Reviews and Meta-Analyses – Scoping Review* (PRISMA-ScR) statement [64] was conducted. To better define the search term text mining algorithms were applied [65]. The search term “big data analytics” was used as a starting point and checked for similarities and thesaurus on *Medline/PubMed* using the search results clustering algorithm Lingo [66,67]. The clustering was based on the first 200 results from a search conducted on April 1^st^, 2019 and revealed overlap of BDA with the terms “predictive analytics”, “advanced analytics”, “machine learning” and “big data analysis methods”.

A combination of these overlapping terms and Boolean operators was used to build the final search term. The search was conducted in *Medline/PubMed* as well as in the computer science database *dblp* (see Table 6 in the appendix). To limit the search results some inclusion and exclusion criteria were applied followed by a qualitative classification of two researchers working independently (see Table 7 in the appendix). For instance articles before 2013 were excluded as the number of articles meeting the inclusion criteria before that date were rather low and Natural Language Processing as a subfield of BDA was excluded because it yielded too much technical articles with few links to integrated care interventions as most often textual information were extracted and analysed from one single source of medical documentation.

To further extract information about strategic interventions in context of the PCIHS framework and about challenges for big data analytics in healthcare content analyses were performed during which the articles chosen for the review were classified (see Tables 8 and 9 in the appendix).

## Results

After elimination of eight duplicates, the search set included 313 articles which were independently categorized by two researchers in “relevant” or “irrelevant” based on titles and abstracts. Disagreements were discussed after the screening process and a consented categorization was agreed upon. This led to 57 articles which were retrieved for full text screening during which two articles were rated as “irrelevant”. The bibliographies of the chosen 55 articles were scanned for a thorough review. Thereby 17 additional publications were added, so that 72 articles were included in the final set (see Figure 4 in the appendix). From the articles in the final set 64% were written by authors in North America, 22% in Europe (incl. UK), 7% in Asia, 3% in the Middle East, 3% in Australia and 1% in Africa (see Table in the appendix). The study type can be broken down in review (33%), case report (24%), quantitative study (18%), technical report (17%), guideline (7%) and survey (1%) (see Table 11 in the appendix). The study settings were scientific research (45%), hospital care (20%), population health management (19%), health insurance (7%), pharmaceutical care (4%), public health (3%) and community care (1%) (see Table 12 in the appendix).

A first and central result of the scoping review was that PCIHS fuel but are also dependent on people-centred models of health data integration and vice versa. If an idealistic model of health service delivery is people-centred and integrated, an idealistic health data analytical platform supporting strategies towards this aim would have to be equally people-centred and integrated. So to answer the research question “How can big data analytics support people-centred and integrated health services?” it seemed helpful to previously develop a role model labelled as people-centred health platform which frames the subsequently presented results of the review. This role model combines the health-related data types across the continuum of care with BDA methods to support the strategies of enabling people-centred care. Which of the data types and analytical methods displayed in the role model are currently used most often in the literature will be presented in the following section. The main research question how BDA can support PCIHS is answered subsequent via the scoping review. Finally, challenges arising from big data analytics in healthcare will be worked out by the content analysis.

### Development of a role model of a people-centred health platform (PCHP)

**Figure 1:**
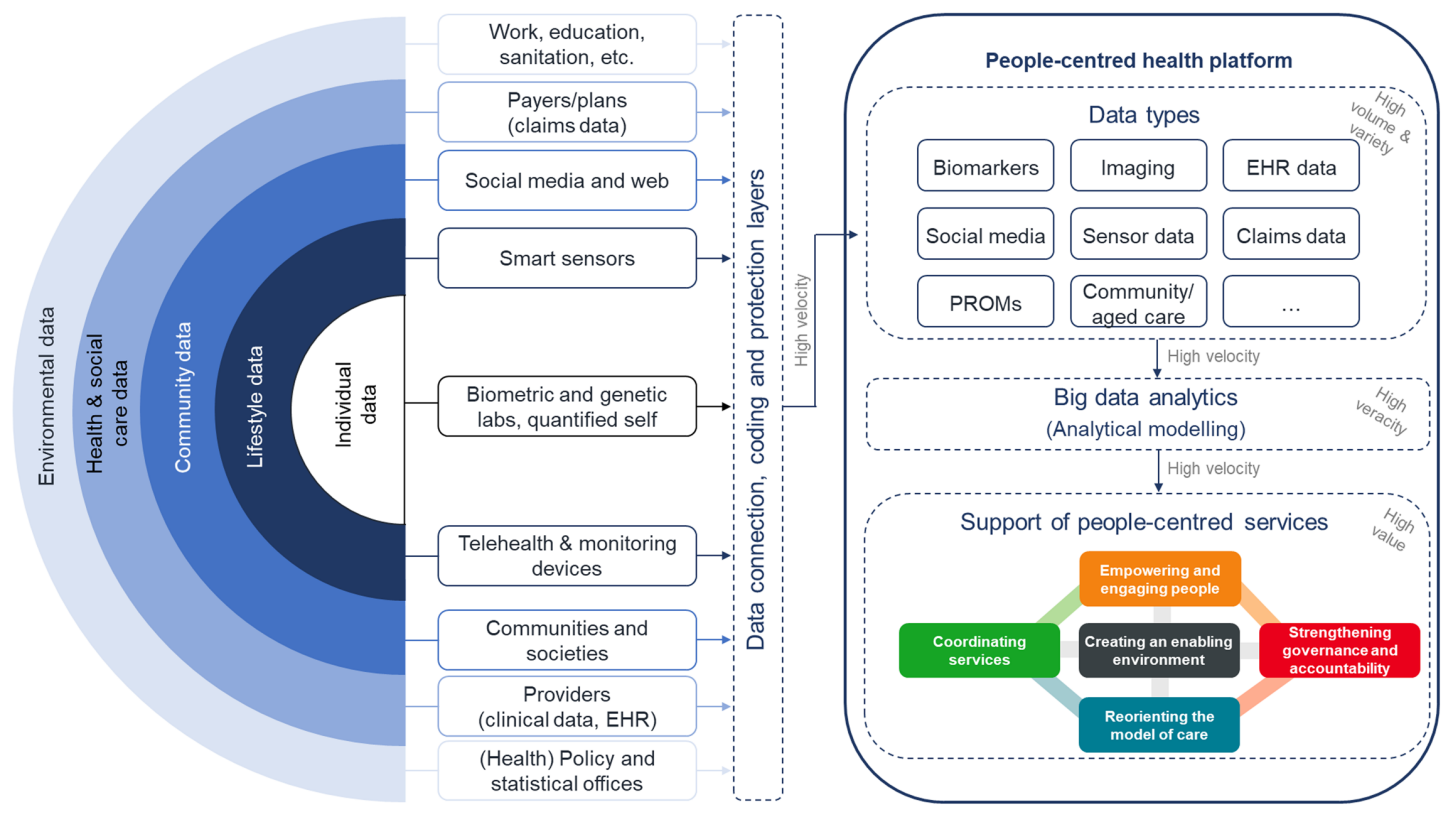
Role model of a people-centred health platform for big data analytics (EHR = electronic health record; PROMs = patient-reported outcome measures, with elements of [37]).

The role model of a people-centred health platform presented in Figure 1 is purposely meant as a roadmap for decision makers to realize data analytical capabilities in healthcare like the PCIHS framework also is an illustration of options healthcare decision makers might consider in optimizing health services dependent on and adapted to their context conditions.

In compliance with the concept of PCIHS all data potentially contributing relevant information about people’s health (rainbow model) were taken into account. Integrating these data in a central health platform as timely as possible (high velocity) would create a data asset of tremendous extent (high volume) and distinctness (high variety). In the data analytics layer big data analytical methods might be applied to the data with the purpose to produce results of high veracity which, interpreted and used by well-informed health decision makers, providers or even patients shall lead to decisions of high value in terms of the five strategies towards people-centred and integrated health services. Comprehensive personal health records are developed and tested by some research institutions [10,53,68,69] as well as in some real-world initiatives such as the national health platforms of Finland [70], Estonia or Australia [71] or from the US Veterans Health Administration [72].

### Types of big data and big data analytical methods in healthcare – Results of the rapid literature review

**Figure 2:**
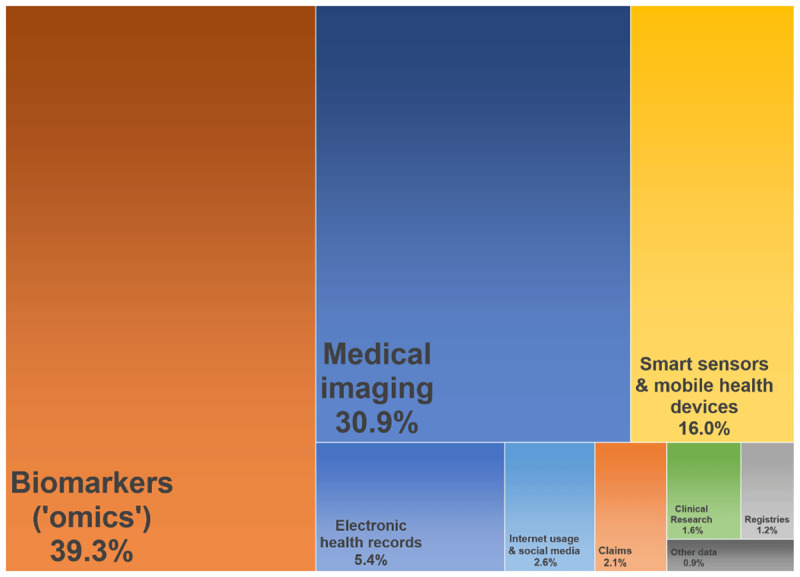
Data types most often applied for big data analyses in healthcare (April 2019), illustrated as tree map.

According to the search results of the rapid literature review biomarker (39.3%) and medical imaging data (30.9%) are currently used most often in publications (see Figure 2). Biomarker data include the whole spectrum of ‘-*omics*’ like genomic, proteomic, or metabolomic data [73,74,75]. Medical images are often part of electronic health records. The most common technologies are ultrasound, computed tomography, magnetic resonance, and x-ray imaging [38,52].

Considerably high rates were also found for smart sensor data (16,0%) and data from electronic health records (5.4%). A smart sensor can be used to constantly track individuals and is often embedded in a smart phone/watch or in telemonitoring devices, sometimes with several devices communicating with each other (*Internet of Things*). A smart sensor can continuously measure large volumes of data in terms of health, fitness, behaviour or lifestyle regardless of location, potentially in real-time and even supplemented by self-reported data (*quantified-self*) [22,27,38,43,74]. A side-specific electronic medical record (EMR) or a cross-institutional electronic health record (EHR) stores data stemming from different source systems which is why technically speaking EMR and EHR are rather data platforms than data types. The volume of data in EHR is massive on the health system level while it varies on the organizational level [37,76]. A typical EHR contains structured data (e.g. medical coding), semi-structured data (e.g. laboratory results) and unstructured data (e.g. narrative clinical notes, medical images) [43,77].

Data types used rather seldom were internet usage or social media data (2.6%), claims data (2.1%, most often health care data, rarely social care data), data from clinical trials (1.6%) and registry data (1.2%). Data generated by using internet technologies include access log data or click streams from websites, search engines, or forums or posts and network relationships from social media platforms or messaging services [22,38,78]. The most common claims data types are medical, pharmaceutical, and ancillary claims while payers hold additional administrative information [26,79]. Claims data are rather homogenous due to specific coding schemes, but at least the data provides a rather full picture of services utilization regardless of the point of care [80], whereas an all-payer database would be ideal for BDA supporting PCIHS so that analytics are not limited to the population covered by a single payer [81].

Other sources like patient surveys, drug surveillance, aged or community care data or other health-related systems together only accounted for less than 1% of current research articles on BDA in healthcare. For example, aged or community care data were presumably underrepresented because most of the provider organization are lacking the financial opportunities to build up and work with large, standardized databases although there would be additional value in using high level information technology and analytics in these contexts [82,83,84]. For PCIHS the integration of as many data sources as possible seems most beneficial.

**Figure 3:**
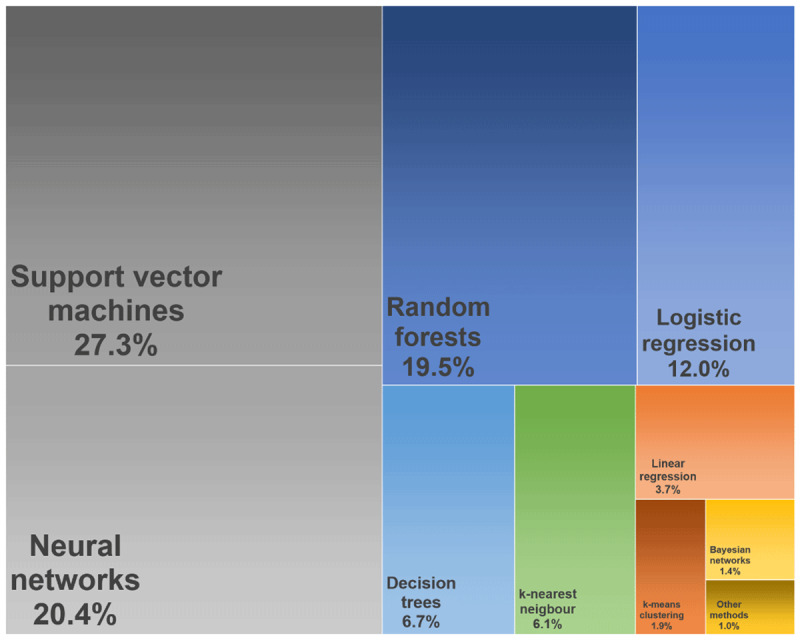
Distribution of the most often used big data analytical models in healthcare (April 2019), illustrated as tree map.

Figure 3 displays the most often used BDA models in healthcare based on the rapid literature review. Support vector machines (27.3%), neural networks (20.4%) and random forests (19.5%) were used most often. Further models used occasionally were decision trees (6.7%), k-nearest neighbour models (6.1%), k-means clustering (1.9%) and Bayesian networks (1.4%). Traditional prediction models in healthcare are primarily parametric regression models based on assumptions regarding the data distribution and a predefined set of input variables [85]. Several studies retrieved in this review labelled their analytics as BDA by applying statistical models to data sources meeting more or less the definition of big data. Therefore considerably high rates were also found for statistical models like logistic regression (12.0%) and linear regression (3.7%) while other methods like multiple regression or proportional hazard models were used rather seldom (~1.0%). The results point to the fact that non-parametric models rather meet the general understanding of BDA in healthcare than traditional statistics.

### How can big data analytics support people-centred health services

A people-centred coordination of preventative, health, and social services (including aged and disability care) is likely impossible without an equally comprehensive integration of the underlying health information technology infrastructure [9,86]. In the scoping literature review articles were screened for analytical applications with the potential to support the five strategies for health services to become more integrated and people-centred. Based on a matrix table (see Table 8 in the appendix) all articles retrieved in the scoping review were categorized with respect to the five strategies of the PCIHS framework or rather to the respective policy options and strategical interventions. The results are summarized in Table 2.

**Table 2:** The strategic interventions of the people-centred and integrated health services framework that might incorporate big data analytics (results of the in this scoping review and a content analysis, see also Table 8).


STRATEGIC DIRECTION	POLICY OPTIONS AND STRATEGICAL INTERVENTIONS POTENTIALLY SUPPORTED BY BDA	NUMBER OF PUBLICATIONS IN THE REVIEW (N = 72)			

**Empowering and engaging people**				**36**	**(51%)**

	Personalized care plans	31	43%		

	Self-management activities	5	7%		

	Shared decision making	4	6%		

	Health education	3	4%		

	Access to personal health records	2	3%		

	Peer support	1	1%		

	Patient satisfaction surveys	1	1%		

**Strengthening governance and accountability**				**23**	**32%**

	Performance evaluation	15	21%		

	Performance-based contracting	8	11%		

	Decentralization	8	11%		

	Patient-reported outcomes	1	1%		

**Reorienting the model of care**				**56**	**79%**

	Clinical decision support	23	32%		

	Tailoring population-based services	19	27%		

	Surveillance and control systems	13	18%		

	Mobile health technologies	10	14%		

	Health promotion and disease prevention	9	13%		

	Home and nursing care	5	7%		

**Coordinating services**				**20**	**28%**

	Care pathways	8	11%		

	Sharing of medical records	6	8%		

	Intersectoral partnerships	5	7%		

	District-based healthcare delivery	1	1%		

**Creating an enabling environment**				**17**	**24%**

	Resource allocation	11	15%		

	System research	6	8%		

	Quality assurance	3	4%		

	Workforce training	2	3%		


### BDA as supporting tool to empower and engage people

At least one of the strategical interventions summarized under empowering and engaging people was named in 36 (51%) of the screened publications. Not only in this domain, but in general the ability of BDA to support the development of personalized care plans was mentioned most often (43%). This could for example be by accurately and timely predicting individual health risks (lifestyle, socio-economics, environment, genetic predisposition, etc.) [26,28,87], by predicting risk scores for disease conversion or progression [8,24], by deciding about the best intervention type based on patient similarity analyses or by predicting the probability for side effects or adverse events [59,88]. Examples found during the review are predictions for chronic diseases, heart failure, type 2 diabetes and severity stages for lung cancer or potential vaccination benefits and risks (see Table 8 for all references). Besides genome-wide association studies uncovering individual genetic predispositions for disease development [93], the full potential of BDA stems from the integration of data on all factors influencing health including also population-based, socio-economic, community-based or environmental factors. By providing information about the likelihood of an individual to benefit from different therapy options more targeted decision aids and medications could be developed and greater satisfaction on the patients’ side be achieved [9,62]. Also, self-diagnostics and self-management activities (7%) could be supported as people could regularly and timely be updated about their situation, their status and their current treatment options [22], e.g. based on sensor or patient-reported data (quantified self) [89]. By sending targeted information accessible via the personal health record or PCHP (3%) the support of peoples health education based on their individual risk factors might be improved (4%), as well as the process of shared decision making (6%) as patients can better define their individual care plans and therefore better adhere to their personal health goals. The PCHP could allow patients not only to access but also to administer and share their health-related data and to use the platform as a tool to communicate e.g. with providers.

### BDA as supporting tool to strengthen governance and accountability

In 23 (32%) of the screened publications BDA was mentioned as a tool to strengthen governance and accountability. BDA could facilitate a deeper understanding of underlying factors for variation across providers, interventions, or regions (appropriate versus avoidable variation) to improve risk adjustment systems or performance evaluations (21%) supporting a transparent competition for outcome improvements [51,80,90], e.g. in performance-based contracts (11%). Also, results could be made publicly available, e.g. in league tables. Geocoded analyses could uncover community-based, regional, or environmental risk factors as well as supplier-induced problems and local disease hot spots [91,92] and be used to establish more decentralized systems (11%) with enhanced scope for local governments or community-care to implement regional health programs enhanced with patient-reported outcomes (1%). This would offer new opportunities for people in local communities to participate in the decision making process via the PCHP as a communication tool and become co-producers of population health.

### BDA as supporting tool to reorient the model of care

The biggest share of articles in the scoping review described potential applications of BDA belonging to the strategy domain of reorienting the model of care (79%). Most often mentioned in this area was incorporating BDA in clinical decision support systems (32%), informing the provider about risks for disease uptake, progression, conversion, decompensation or the development of comorbidities [58,93]. A key factor of the PCIHS strategy of reorienting the model of care is strengthening primary and community care, whereas BDA could support more accurate diagnostics at these points of care [51,94,95,96]. Clinical judgements in these sectors might e.g. benefit from proactive alerts which inform about individual risks for preventable events like (re-)admissions to hospital, for intensified resource use, for (post-surgical) complications or disease progression [93,94,95,96], in the best case based on intersectoral health data from the PCHP also allowing for interdisciplinary communication. According to a survey in the USA, 15% of the healthcare providers already have access to some kind of predictive analytics and the conditions most often targeted were hospital readmissions (27%), the development of a sepsis (27%), patient deterioration (18%) and general health (10%) [97]. Using intersectoral data to stratify individuals into (chronic) care groups and identify comparable or manageable populations could support additional population health management activities (26%) in which the role of nurses and community health workers could be enhanced [22,24,26,35,98]. Also surveillance and control systems (18%) could benefit from BDA based on real world health data assets, e.g. the surveillance of adverse drug and vaccination effects or the monitoring of disease transmission patterns or outspread speed of epidemics or pandemics [91,92] enabling for example faster reaction and better targeted campaigns [88]. Using real-world data would additionally allow for rather small risk groups or (geographically) isolated communities already suffering from under-coordination to also be taken into consideration in healthcare decision making [44,99]. Furthermore, activities like health promotion and disease prevention (13%) might be better tailored to individuals if certain risk factors are specifically addressed. By using sensing devices as well as mobile technologies (14%) or devices within the patients’ ambient (6%) therapy results might be better tracked by patients as well as by providers.

### BDA as supporting tool to coordinate services within and across sectors

In the scoping review 20 publications (28%) described BDA as a tool to support service coordination. Most articles mentioned the development and evaluation of intersectoral care pathways (11%) by exploring comparable patterns and then setting up multidisciplinary task forces of medical and non-medical providers for such multi-layered problems structured around an individual’s social experiences and comorbidities. Also, BDA respective the PCHP as enabler would simplify the exchange of medical records (8%), especially in the transition between hospital and home. Four publications (6%) described BDA as an enabling tool for intersectoral partnerships across the health sector (e.g. with social security, housing, education) to provide holistic care and one publication described a model in which BDA is used for district-based healthcare delivery [100].

### BDA supporting the creation of an enabling environment

The strategy of creating an enabling environment is supporting the aforementioned strategies and is rather broad in scope. BDA itself is an enabler for people-centred health services, but 17 publications (24%) mentioned BDA as incorporated in other enabling factors as well. On the level of resource planning and allocation (15%) BDA might be capable of reducing financial waste by identifying common patterns of fraud and abuse or by uncovering disincentives of the renumeration system towards finding the right payment mix [79,80,101]. BDA could also support system research comparing the effects of different system architectures (9%). Assisting in quality assurance (4%), BDA could, e.g. by exploring care patterns, identify clinical waste and provide the opportunity to get rid of ineffective or unnecessary interventions or to reduce over- and undertreatment [37,44]. Two publications described BDA as tool to identify those professionals benefitting the most from additional training and education, e.g. on team-based culture or open feedback (3%).

### Challenges of big data analytics in healthcare discussed in the literature

As BDA has the potential to improve PCIHS it seems valuable to find solutions for the challenges stemming from big data in healthcare [102]. Currently the situation for most stakeholders is characterized by confusion or uncertainty [54]. Of the 72 articles in this review, 45 (62.5%) discussed at least one BDA challenge. Most often discussed were methodological challenges (54.2%) followed by regulatory (43.1%) and technological challenges (41.7%). Cultural challenges were less often discussed (25.0%). The five issues mentioned most often in making better use of BDA were missing modelling standards and potential bias (36.1%), a questionable evidence-base of BDA results (33.3%), poor data quality (27.8%), the lack of an appropriate framework for privacy protection (26.4%) and the lack of interoperability requirements for data linkage (26.4%). In the successive descriptions only the most relevant publications will be referenced (see Table 9 in the appendix for more details).

### Regulatory challenges

From a regulatory perspective it is challenging to set up a framework to coordinate, support and financially incentivize the efforts in building a big data platform for health data [15]. Besides ensuring for targeted investments this means describing the policies of appropriate data storage [27,36]. As the relevance of analytical results in clinical processes diminishes over time it is also a challenge to facilitate user friendly processes for data entry and timely exchange to finally enable (real-time) recommendations at the point of care [9,36,103]. To overcome legal or commercial barriers across domains intellectual property rights must be clearly defined, penalizing e.g. the unwillingness to share relevant (clinical) data for economic reasons or unintended uses [41]. To avoid underperforming models from mis-informing clinical decision making, a framework for transparent model development and evaluation would be needed [46,104,105]. Analytical modelling standards could, comparable to drug licensing, be transparently developed by quality controlled institutions which incorporate the technical and methodological expertise but also contribute domain knowledge to determine how to provide accurate, reliable and actionable information for patient care [44,106]. Likewise, this is touching ethical issues, e.g. if a BDA model at the beginning of the learning curve provides seriously harmful recommendations for some individuals [88,107,108]. The most often mentioned regulatory challenge was the design of an appropriate framework finding the sweet spot between transparency and protecting privacy enabling as effective decision supporting analytics as possible without enabling a potentially manipulative misuse of the data [54,77]. To enable as many beneficial analytics as possible, it might be an option to make deidentified data extracts from the PCHP accessible for chosen academic or even commercial purposes [9,54,77].

### Technological challenges

Despite prices for data storage are steadily going down from a technological perspective the design of an infrastructure appropriate for storing and curating massive amounts of diverse health data is still a complex task [37,38,77,108]. Also, it is challenging to deal with high-velocity data depending on considerable computational processing resources and then to use appropriate software tools for data analytics [27,85]. Blending the extremely diverse and often unstructured health data from heterogenous sources leads to the challenge of establishing technological standards of interoperability [77,89]. Furthermore, inaccurately calibrated measurement systems as well as hard- and software failures (e.g., wrong auto-fill-in functions) inadequate data transfer protocols or not adequately developed software pose risks for data quality. Data quality problems can possibly arise at every step during data generation while the chance for bias might be lower for recorded medical signals than for manually documented features [36,39,54]. Finally, all layers of a big data platform (storage, transfer, analytics, presentation) have to be technically protected against unintended uses or breaches, e.g. by data encryption, certification or access authentication [72,77]. Big data technologies were out of the scope of this review, but at least it shall be referenced to articles discussing tools for big data storage & transformation like MongoDB or Apache HBase [9,38,43,74,108], for big data processing & analysing like Hadoop or MapReduce [38,43,74,85,109] as well as methods for (big) data security [77,110,111,112].

### Methodological challenges

From a methodological perspective it is challenging to work on a high-dimensional database likely to contain more feature variables than observable subjects [44] and to develop real-time analytical models as most documentation processes in healthcare traditionally are rather slow [36,72]. Regarding human documentation also data entry errors like incomplete, incongruent, or missing data and a poor update status pose risks for data quality [39]. As a priori it is unclear which model is most appropriate for the targeted type of application and which model offers clinically more meaningful interpretations, the process of evaluating analytical models is quite challenging [113]. It affects the analytical results that no commonly accepted methodological standard for modelling exists offering nearly unlimited different options for the combination of variables whereas currently there is a lack of knowledge about which methods to use for which purposes and the black box design of some machine learning algorithms even exacerbates their comprehensibility. Additionally, external validity or generalizability is a challenge as it is difficult to compare the performance of different BDA models based on different data types from different regions [77,113,114]. It is also problematic that in some source systems data is recorded for specific reasons (e.g., medical billing) or with different coding standards potentially limiting interpretability beyond the original purpose. In a greater extent the same limitations as for observational studies also apply for BDA such that it is extremely difficult to exclude potential bias (e.g. selection bias, confounding bias, measurement bias), that due to missing randomization no causal relationships can be determined and that especially BDA has a high risk for modelling artefacts like random noise or overfitting [27,56,87]. Designing a methodology on how to evaluate the clinical usefulness and evidence-base of the analytical models or their effectiveness and safety in part also is a methodological issue [115]. To date, there is only minimal evidence that BDA in healthcare revealed anything surprisingly new and can effectively improve decision making or medical outcomes [93,94,116]. Furthermore, is has not been proven that machine learning models outperform traditional statistical models in predictive or exploratory tasks. Most often only sparse differences in the model performance are observed, maybe because they were often applied to rather small data sets limiting the ability of BDA models to optimize the inductive feature selection process [7,8,113]. To disseminate information about the most effective treatments to the intended providers at the point of care requires that information overload is prevented, and analytical results are timely and easily accessible, appropriately simplified, appealingly visualized and well-integrated in clinical workflows [93,117]. A comprehensive discussions of methodological issues of BDA in healthcare is e.g. provided by Hoffman/Podgurski [54] and Van Poucke et al. [46].

### Cultural challenges

An adaption of BDA models in healthcare requires appropriate education as well as a shift towards team-based analytics enhancing medical domain knowledge with skills e.g. from data science and health economics [37,85]. Form an organizational perspective also resistances against expanding and speeding-up electronic data exchange and against redesigning clinical workflows with data-driven feedback need to be overcome by communicating potential benefits and by putting media-hyped expectations into perspective [25,72]. A data quality culture must be developed to reduce behaviours like unreflective copy-pasting and strategical manipulation of data. From the societal perspective, a data sharing culture would be helpful to counteract personal and organizational concerns. This might be accompanied by an open science culture which ensures that peoples’ data are used as intended [22,36,118]. Exploratory studies point to the fact that the majority of people is willing to share health data for population-based health research, but fewer individuals are comfortable to have their data used to improve medical decision making or to adapt insurance rates [119,120] with country-specific, cultural differences [121,122]. As the mere existence of BDA tools does not influence value improvement a learning culture with engaged providers needs to be achieved with (clinical) usability as a precondition.

In Table 3 all challenges mentioned above were systematized by combining the domains of technological, methodological, regulatory, and cultural challenges [37,74] with the 5-V model as each big data characteristic entails specific obstacles [36,54,77,85].

**Table 3:** Challenges in designing a people-centred and integrated health platform to enable big data analytics in healthcare.


CHALLENGE DOMAIN BIG DATA CHARACTERISTIC	REGULATORY	TECHNOLOGICAL	METHODOLOGICAL	CULTURAL

**Volume**	Investment & technology framework	Data infrastructure	High-dimensional analytics	Teamwork culture

**Velocity**	Communication framework	Data processing	Real-time analytics	Delivery process redesign

**Variety**	Intellectual property framework	Data linkage	Modelling standards & bias	Data sharing culture

**Veracity**	Evaluation framework	Data quality	Evidence- base	Data governance

**Value**	Privacy & ethics framework	Data access & data security	Interpretation & usability	Culture of learning & change


Potential success factors of big data analytics or strategies to overcome the challenges can be derived as countermovement to each challenge displayed in Table 3. For example the success factor of data quality assurance would be a strategical reaction to the described data quality challenges as well as the success factor of implementing a big data governance would be a reaction to the fact that healthcare organizations are often missing a data governance. The enabling factors of the PCIHS framework [32] as well as some articles from the scoping review provide further information [105,123].

## Limitations

The results presented in this article depend on the literature found by using the defined search terms and also depend on the timing of the literature review. Although text mining algorithms were applied to refine the search terms it may be that a subclass of potentially relevant articles was not covered because domain-specific words were used or that relevant articles were unintentionally excluded by the exclusion criteria. If further literature databases as well as other languages than English or such literature being published between conduction and publication of this review were also included in the review, this would have enhanced the number of articles. As indicated by the frequency distribution of the authors’ country affiliation, experiences of middle- and low-income countries seem underrepresented. And also from high-income countries it may be that there is a certain number of data analytical applications nothing has been published about yet. The topic of Natural Language Processing (NLP) was intentionally excluded which does not mean that is does not also pose potential in supporting integrated care activities. Publication bias might have limited the results to scientifically relevant articles on rather novel topics, on articles with rather positive outcomes or on health-related issues where large databases already exist. Therefore, in the results part, data types and areas of applications are highlighted which were already described by researchers performing big data analytics, while areas of application, for which large datasets do not exist to the same extend (e.g., for social care, public health or preventative care, community care, education, or disability services) were underrepresented. Quite the opposite does this mean that additional data analytics might have less potential value, but rather that the source systems need to be further developed to be suitable for big data analytics. For some important components of the framework on people-centred care like enhancing the role of community care or establishing intersectoral partnerships between health and social care only few examples of enabling big data analytical tools were found in the literature.

## Conclusion and outlook

This review aimed to make a contribution to the research question “How can big data analytics support people-centred and integrated health services”. The role model of the people-centred health platform may in combination with the PCIHS framework be used by health policy and healthcare decision makers as a design principle to guide (national) strategies, whereas no universally valid approach that can be applied in all contexts. Rather should the strategical options and potentials gathered be prioritized with respect to the specific circumstances and financial opportunities to enable developments in the desired direction. The BDA methods and practical applications have a tremendous potential to improve integrated care interventions with respect to better health quality and efficiency and at least the methods can already be incorporated by health professionals or health management organizations. But it has also to be stated that up to now big data analytics does not fulfil the oversized expectations and already constitutes better outcome with respect to the triple aim. Likely this is because health-related data is extremely sensitive and complex and there are few practical examples of data platforms to some extent already capable of merging and providing people-centred big data so that the models and applications described in this work cannot evolve their full potential. But anyhow the integration of health data can be expected to further proceed. Every foreseeable integration of health data – e.g., genetic data in electronic health records – is at least a small step to also improve people-centred care and in the near future these sources will be merged with additional health-related data types on individual level. It might be a long way until BDA enable a faster reaction on dynamic situations like pandemics, a more need-based distribution of resources across the continuum of care and a more detailed understanding of the complex factors that have an impact on individual and population-based health but although the challenges are big and efforts are high this movement will further proceed as the potential benefits cannot be neglected.

## Additional File

The additional file for this article can be found as follows:

10.5334/ijic.5543.s1 Appendix and Search terms and data. Figure 4 and Tables 4 to 12.
