# Supplementary material for: How can Big Data Analytics Support People-Centred and Integrated Health Services: A Scoping Review
Source: Int J Integr Care. 2022 Jun 16;22(2):23. doi: 10.5334/ijic.5543 (PMC9205381; doi:10.5334/ijic.5543)
Supplement: Appendix and Search terms and data. — Figure 4 and Tables 4 to 12. [file ijic-22-2-5543-s1.pdf]

Appendix

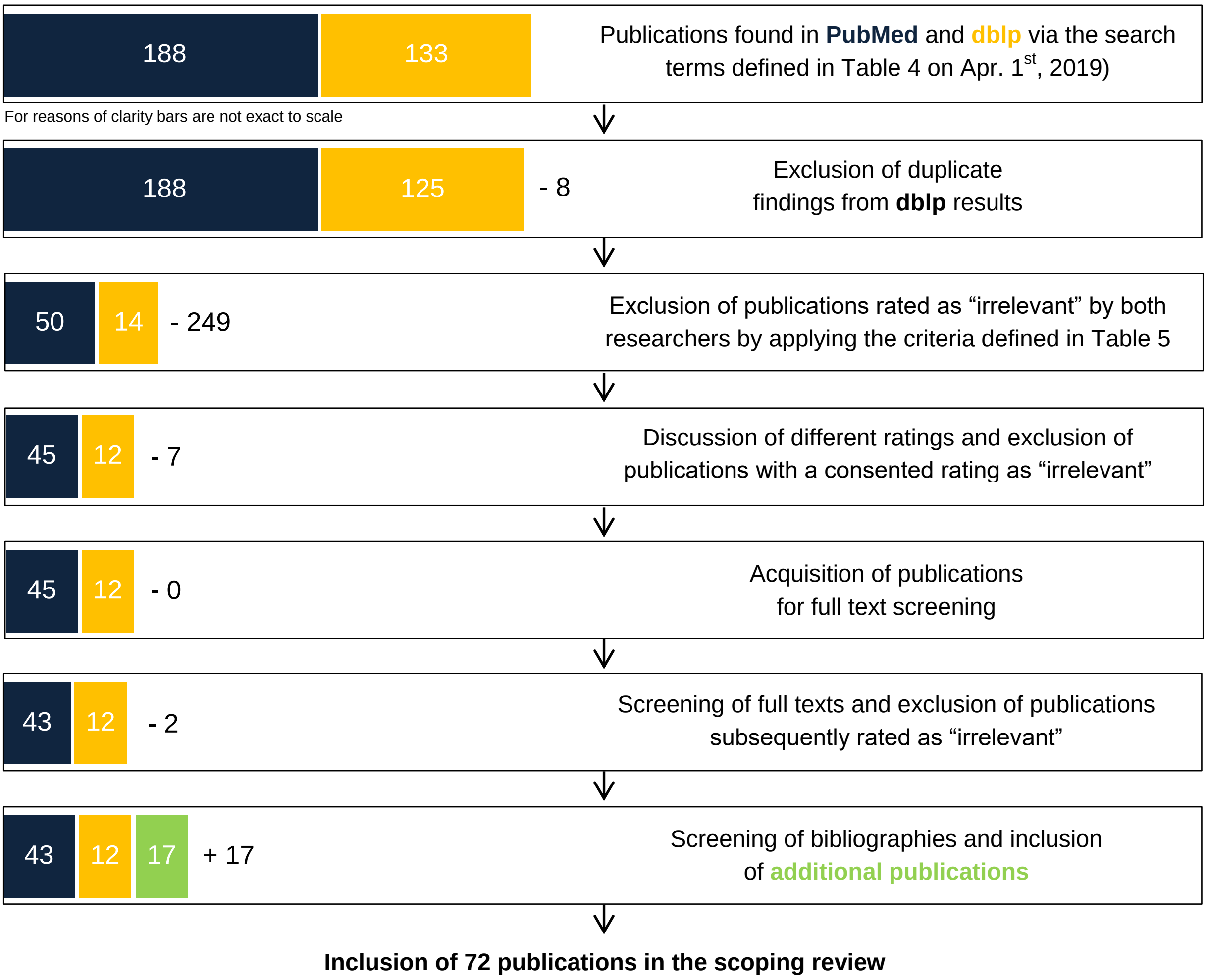

Figure 4: Graphical representation of the scoping review process

Search terms and data

Database	Data type	Search term	Language	Search Fields	Limiters	Search results (Apr. 1st, 2019)	Proportion
Pub Med	Electronic health record data	(big data analy* [Title/Abstract] OR advanced analy* [Title/Abstract] OR predictive analy* [Title/Abstract] OR machine learning [Title/Abstract]) AND (EHR [Title/Abstract] OR EMR [Title/Abstract] OR electronic health record* [Title/Abstract] OR electronic medical record* [Title/Abstract]))	English	Title/ Abstract	Journals	467	5.45%
Pub Med	Transactional claims data	((big data analy* [Title/Abstract] OR advanced analy* [Title/Abstract] OR predictive analy* [Title/Abstract] OR machine learning [Title/Abstract]) AND (claim* [Title/Abstract] OR administrative data [Title/Abstract] OR transactions data [Title/Abstract] OR billing data [Title/Abstract] OR health business data [Title/Abstract]))	English	Title/ Abstract	Journals	178	2.08%
Pub Med	Data from clinical registries	((big data analy* [Title/Abstract] OR advanced analy* [Title/Abstract] OR predictive analy* [Title/Abstract] OR machine learning [Title/Abstract]) AND (registry [Title/Abstract] OR registries [Title/Abstract]))	English	Title/ Abstract	Journals	99	1.16%
Pub Med	Smart sensor and smart device data	((big data analy* [Title/Abstract] OR advanced analy* [Title/Abstract] OR predictive analy* [Title/Abstract] OR machine learning [Title/Abstract]) AND (device* [Title/Abstract] OR sensor* [Title/Abstract] OR smartphone* [Title/Abstract] OR wearable* [Title/Abstract]))	English	Title/ Abstract	Journals	1,370	15.99%
Pub Med	Internet usage and social media data	((big data analy* [Title/Abstract] OR advanced analy* [Title/Abstract] OR predictive analy* [Title/Abstract] OR machine learning [Title/Abstract])	English	Title/ Abstract	Journals	223	2.60%

		AND (social media [Title/Abstract]<br>OR web search [Title/Abstract]<br>OR health portal [Title/Abstract]<br>OR health information [Title/Abstract]))					
Pub Med	Data from clinical trials	((big data analy* [Title/Abstract]<br>OR advanced analy* [Title/Abstract]<br>OR predictive analy* [Title/Abstract]<br>OR machine learning [Title/Abstract])<br>AND (clinical trial [Title/Abstract]<br>OR RCT [Title/Abstract]<br>OR controlled trial [Title/Abstract]))	English	Title/ Abstract	Journals	137	1.60%
Pub Med	Imaging data	((big data analy* [Title/Abstract]<br>OR advanced analy* [Title/Abstract]<br>OR predictive analy* [Title/Abstract]<br>OR machine learning [Title/Abstract])<br>AND (*imaging [Title/Abstract]<br>OR images [Title/Abstract]))	English	Title/ Abstract	Journals	2,644	30.86%
Pub Med	Biomarker data	((big data analy* [Title/Abstract]<br>OR advanced analy* [Title/Abstract]<br>OR predictive analy* [Title/Abstract]<br>OR machine learning [Title/Abstract])<br>AND (omics [Title/Abstract]<br>OR gene [Title/Abstract]<br>OR genetic [Title/Abstract]<br>OR DNA [Title/Abstract]<br>OR Biomarker [Title/Abstract]))	English	Title/ Abstract	Journals	3,370	39.34%
Pub Med	Health-related systems data	((big data analy* [Title/Abstract]<br>OR advanced analy* [Title/Abstract]<br>OR predictive analy* [Title/Abstract]<br>OR machine learning [Title/Abstract])<br>AND (socio-economic data [Title/Abstract]<br>OR census [Title/Abstract]<br>OR location data [Title/Abstract]<br>OR environmental data [Title/Abstract]))	English	Title/ Abstract	Journals	52	0.61%
Pub Med	Patient-reported outcomes and survey data	((big data analy* [Title/Abstract]<br>OR advanced analy* [Title/Abstract]<br>OR predictive analy* [Title/Abstract]<br>OR machine learning [Title/Abstract])<br>AND (patient reported outcome [Title/Abstract]	English	Title/ Abstract	Journals	27	0,32%

		OR PROM [Title/Abstract] OR survey data [Title/Abstract]))					

Table 4: Search terms for the rapid review on big data types in healthcare

Database	Data type	Search term	Language	Search Fields	Limiters	Search results (Apr. 1st, 2019)	Proportion
PubMed	Logistic regression	((big data analy* [Title/Abstract] OR advanced analy* [Title/Abstract] OR predictive analy* [Title/Abstract] OR machine learning [Title/Abstract]) AND ("logistic regression" [Title/Abstract]))	English	Title/ Abstract	Journals	1,026	12.03%
PubMed	Linear regression	((big data analy* [Title/Abstract] OR advanced analy* [Title/Abstract] OR predictive analy* [Title/Abstract] OR machine learning [Title/Abstract]) AND ("linear regression" [Title/Abstract]))	English	Title/ Abstract	Journals	312	3.66%
PubMed	Neural network	((big data analy* [Title/Abstract] OR advanced analy* [Title/Abstract] OR predictive analy* [Title/Abstract] OR machine learning [Title/Abstract]) AND ("neural network" [Title/Abstract]))	English	Title/ Abstract	Journals	1,742	20.43%
PubMed	Bayesian network	((big data analy* [Title/Abstract] OR advanced analy* [Title/Abstract] OR predictive analy* [Title/Abstract] OR machine learning [Title/Abstract]) AND ("Bayesian network" [Title/Abstract]))	English	Title/ Abstract	Journals	123	1.44%
PubMed	Decision tree	((big data analy* [Title/Abstract] OR advanced analy* [Title/Abstract] OR predictive analy* [Title/Abstract] OR machine learning [Title/Abstract]) AND ("decision tree" [Title/Abstract]))	English	Title/ Abstract	Journals	569	6.67%
PubMed	k-nearest neighbour	((big data analy* [Title/Abstract] OR advanced analy* [Title/Abstract] OR predictive analy* [Title/Abstract] OR machine learning [Title/Abstract]) AND ("k-nearest" [Title/Abstract]))	English	Title/ Abstract	Journals	517	6.06%
PubMed	Random forest	((big data analy* [Title/Abstract] OR advanced analy* [Title/Abstract]	English	Title/ Abstract	Journals	1,664	19.51%

		OR predictive analy* [Title/Abstract]<br>OR machine learning [Title/Abstract])<br>AND ("random forest" [Title/Abstract]))					
PubMed	k-means<br>clustering	((big data analy* [Title/Abstract]<br>OR advanced analy* [Title/Abstract]<br>OR predictive analy* [Title/Abstract]<br>OR machine learning [Title/Abstract])<br>AND ("k-means" [Title/Abstract]))	English	Title/<br>Abstract	Journals	163	1.91%
PubMed	Support vector machine	((big data analy* [Title/Abstract]<br>OR advanced analy* [Title/Abstract]<br>OR predictive analy* [Title/Abstract]<br>OR machine learning [Title/Abstract])<br>AND ("support vector machine" [Title/Abstract]))	English	Title/<br>Abstract	Journals	2,327	27.29%
PubMed	Regularized regression	((big data analy* [Title/Abstract]<br>OR advanced analy* [Title/Abstract]<br>OR predictive analy* [Title/Abstract]<br>OR machine learning [Title/Abstract])<br>AND ("regularized regression" [Title/Abstract]))	English	Title/<br>Abstract	Journals	22	0.26%
PubMed	Multiple<br>regression	((big data analy* [Title/Abstract]<br>OR advanced analy* [Title/Abstract]<br>OR predictive analy* [Title/Abstract]<br>OR machine learning [Title/Abstract])<br>AND ("multiple regression" [Title/Abstract]))	English	Title/<br>Abstract	Journals	43	0.50%
PubMed	Proportional hazard model	((big data analy* [Title/Abstract]<br>OR advanced analy* [Title/Abstract]<br>OR predictive analy* [Title/Abstract]<br>OR machine learning [Title/Abstract])<br>AND ("proportional hazard" [Title/Abstract]))	English	Title/<br>Abstract	Journals	19	0,22%

Table 5: Search terms for the rapid review on analytical methods for BDA in healthcare

Database	Search term	Language	Search Fields	Limiters	Search results (Apr. 1st, 2019)
PubMed	(<br>big data analy*[Title/Abstract]<br>OR advanced analy*[Title/Abstract]<br>OR predictive analy*[Title/Abstract]<br>OR (machine learning [Title/Abstract]	English	Title/<br>Abstract	Case report, guideline,<br>observational study,<br>review,	188

	AND<br>observational [Title/Abstract])<br>)<br>NOT (genetic* [Title/Abstract]<br>OR chemic* [Title/Abstract]<br>OR biologic* [Title/Abstract]<br>OR *omic* [Title/Abstract]<br>OR molecul* [title/abstract]<br>OR clinical trial [Title/Abstract]<br>OR natural language<br>processing [Title/Abstract]<br>)			systematic review,<br>technical report	
dblp	Big data analy* health<br>Advanced analy* health<br>Predictive analy* health<br>Visual analy* health<br>Machine learning health	English	All	Journal article	133

Table 6: Final search terms for the scoping review

**Criteria**	**Included**	**Excluded**
Dates	≥ 2013	< 2013
Language	English	All other languages
Article type	(Systematic) reviews, case studies, discussion papers, guidelines, method papers, technical reports, research papers with BDA application	Books, qualitative research, clinical trials, editorials, conference supplements, research papers without BDA application, etc.
Content	- big data platform<br>- big data technologies<br>- health data types/sources<br>- analytical models for big data<br>- BDA applications/use cases<br>- BDA challenges/ potentials	- Natural language processing<br>- big data only in the outlook<br>- genetic/molecular/etc. research<br>- no big data and no advanced analytics<br>- too specific, not directly human health-related context, e.g. BDA for animal research, crop science or air pollution
Filter	machine learning for observational research	machine learning in general as most often used in genomic studies

Table 7: Inclusion and exclusion criteria for the scoping review

**Strategy**	**Strategic Intervention**	**References mentioning BDA as supporting tool**	**Number and % of**

domain			References (n= 72)	
Empowering and engaging people	Personalized care plans	[8–10, 14, 15, 24, 26, 28, 40, 42–44, 46, 47, 51, 52, 56, 59, 72, 74, 75, 87–89, 93–95, 98, 104, 116, 124]	31	43.1%
	Self-management activities	[22, 43, 44, 50, 74]	5	6.9%
	Shared decision making	[40, 51, 105, 125]	4	5.6%
	Health education	[15, 46, 89]	3	4.2%
	Access to personal health records	[71, 105]	2	2.8%
	Peer support	[43]	1	1.4%
	Patient satisfaction surveys	[74]	1	1.4%
Strengthening governance and accountability	Performance evaluation	[10, 26, 27, 45, 46, 54, 74, 79, 91, 93, 98, 100, 101, 103, 104]	15	20.8%
	Performance-based contracting	[26, 27, 45, 79, 93, 98, 101, 104]	8	11,1%
	Decentralization	[9, 43, 74, 92, 93, 100, 124, 125]	8	11,1%
	Patient-reported outcomes	[51]	1	1.4%
Reorienting the model of care	Clinical decision support	[7, 10, 14, 15, 24, 26, 27, 36, 40, 46, 48, 58, 59, 74, 76, 87, 90, 93, 95, 105, 114, 126, 127]	23	31.9%
	Population-based services	[8, 14, 15, 22, 24, 26, 27, 46, 52, 56, 59, 61, 80, 87, 91, 93, 94, 124, 128]	19	26.4%
	Surveillance and control systems	[8, 14, 43, 44, 46, 47, 54, 58, 74, 88, 91, 93, 125]	13	18.1%
	Mobile health technologies	[7, 15, 38, 40, 41, 71, 74, 89, 104, 108]	10	13.9%
	Health promotion and disease prevention	[22, 28, 51, 85, 89, 98, 115, 116, 129]	9	12.5%
	Home and nursing care	[14, 50, 60, 72, 89]	5	6.9%
Coordinating services	Care pathways	[22, 24, 61–63, 80, 124, 128]	8	11,1%
	Sharing of medical records	[10, 41, 71, 76, 77, 105]	6	8.3%
	Intersectoral partnerships	[10, 45, 50, 74, 116]	5	6.9%
	District-based healthcare delivery	[100]	1	1.4%
Creating an enabling environment	Resource allocation	[15, 37, 38, 40, 45, 51, 54, 79, 101, 105, 108]	11	15.3%
	System research	[9, 46, 50, 75, 93, 104]	6	8.3%

	Quality assurance	[14, 39, 113]	3	4.2%
	Workforce training	[74, 106]	2	2.8%

Table 8: Matrix table on the strategic interventions of the people-centred and integrated health services framework supported by big data analytics in this scoping review

**Challenge domain**	**Challenge**	**References mentioning BDA challenges (n = 45 (63.3%) of 71)**	**Number and % of references (n = 72)**	
Regulatory	Investment framework	[7, 27, 36, 58, 77, 85, 104]	7	9.7%
	Communication framework	[9, 36, 41, 103–105]	6	8.3%
	Intellectual property framework	[9, 14, 15, 36, 39, 41, 44, 72, 77, 88, 108]	11	15.3%
	Evaluation framework	[41, 46, 52, 72, 75, 76, 85, 104–106, 115, 116, 124]	13	18.1%
	Privacy framework	[14, 15, 27, 35–37, 41, 44, 46, 52, 54, 71, 75, 77, 85, 88, 89, 93, 105, 124]	20	27.8%
	Ethics framework	[7, 41, 88, 104, 108]	5	6.9%
Technological	Data infrastructure	[9, 15, 27, 36–38, 46, 59, 75, 77, 85, 89, 104, 108]	14	19.4%
	Data processing	[9, 14, 27, 28, 36, 38, 46, 59, 72, 75, 88, 104, 108, 124]	14	19.4%
	Data linkage	[14, 27, 28, 36–39, 44, 46, 54, 72, 77, 85, 88, 89, 104, 108, 116, 124]	19	26.4%
	Data quality	[7, 10, 14, 27, 37–39, 41, 44, 46, 54, 72, 75, 77, 85, 93, 104, 108, 125]	19	26.4%
	Data access & security	[9, 14, 15, 27, 37, 38, 44, 46, 52, 54, 71, 72, 75, 77, 88, 93, 108, 124]	18	25.0%
Methodological	High-dimensional analytics	[9, 44, 46]	3	4.2%
	Real-time analytics	[14, 36, 38, 44, 72, 75, 77, 85, 88, 89, 104, 105, 124, 127]	14	19.4%
	Modelling standards & bias	[7, 10, 14, 27, 36, 39, 44, 46, 54, 59, 72, 75, 77, 85, 87, 89, 90, 92, 93, 104, 108, 113–115, 124, 125]	26	36.1%
	Evidence-base	[7, 9, 27, 36, 39, 41, 44, 46, 48, 52, 54, 56, 58, 72, 75–77, 89, 93, 104, 115, 116, 124, 125]	24	33.3%
	Interpretation & usability	[7, 14, 27, 46, 48, 58, 59, 71, 72, 85, 90, 91, 93, 104, 115, 124, 125]	17	23.6%
Cultural	Teamwork culture	[9, 27, 36, 37, 46, 58, 59, 72, 75, 85, 89, 105]	12	16.7%
	Process redesign	[15, 27, 36, 40, 46, 72, 105]	7	9.7%
	Data sharing culture	[15, 36, 41, 44]	4	5.6%
	Data governance	[27, 72]	2	2.8%
	Learning & change culture	[9, 27, 37, 58, 72, 77, 85, 93, 105]	9	12.5%

Table 9: Main challenges of BDA discussed in the screened literature

**Country assignment of the first author**	**Number of publications in the review (n = 72)**
USA	*43*
Canada	*3*
United Kingdom	*3*
Australia	*2*
China	*2*
Denmark	*2*
Germany	*2*
Saudi Arabia	*2*
Spain	*2*
Switzerland	*2*
Austria	*1*
Belgium	*1*
India	*1*
Italy	*1*
Malaysia	*1*
Poland	*1*
South Africa	*1*
Sweden	*1*
Turkey	*1*

Table 10: Publications included in the scoping review by country

**Study type**	**Number of publications in the review (n = 72)**
Review	*24*
Case report	*17*
Quantitative analysis	*13*
Technical report	*12*
Guideline	*5*
Survey	*1*

Table 11: Publications included in the scoping review by study type

**Research setting**	**Number of publications in the review (n = 72)**
Research	*34*
Hospital	*15*
Population health management	*14*
Health insurance	*5*
Pharmaceuticals	*4*

Public health	*2*
Community care	*1*

Table 12: Publications included in the scoping review by setting

References

124. Belle A, Thiagarajan R, Soroushmehr SMR, et al. Big data analytics in healthcare. BioMed Research International. 2015; 1–16. DOI: https://doi.org/10.1155/2015/370194

125. Bragazzi NL, Alicino C, Trucchi C, et al. Global reaction to the recent outbreaks of Zika virus: insights from a big data analysis. PLOS ONE. 2017; 12(9): e0185263. DOI: https://doi.org/10.1371/journal.pone.0185263

126. Kaewprag P, Newton C, Vermillion B, et al. Predictive models for pressure ulcers from intensive care unit electronic health records using Bayesian networks. BMC Med Inform Decis Mak; 2017. DOI: https://doi.org/10.1186/s12911-017-0471-z

127. Taylor RA, Pare JR, Venkatesh AK, et al. Prediction of in-hospital mortality in emergency department patients with sepsis: a local big data-driven, machine learning approach. Academic Emergency Medicine. 2016; 23(3): 269–278. DOI: https://doi.org/10.1111/acem.12876

128. Makar M, Ghassemi M, Cutler DM, Obermeyer Z. Short-term mortality prediction for elderly patients using medicare claims data. International Journal of Machine Learning and Computing. 2015; 5(3): 192–197. DOI: https://doi.org/10.7763/IJMLC.2015.V5.506

129. Bihorac A, Ozrazgat-Baslanti T, Ebadi A, et al. MySurgeryRisk: development and validation of a machine-learning risk algorithm for major complications and death after surgery. Annals of Surgery. 2018; 1. DOI: https://doi.org/10.1097/SLA.0000000000002706
